# Practical application of laser reflectance confocal microscopy in the follow-up of patients with lentigo maligna undergoing treatment with Imiquimod^⋆^

**DOI:** 10.1016/j.abd.2021.01.008

**Published:** 2022-06-09

**Authors:** Priscila Ishioka, Lilian Lemos Costa, Marcus Maia

**Affiliations:** a Dermatology Oncology Outpatient Clinic, Department of Internal Medicine, Irmandade da Santa Casa de Misericórdia de São Paulo, São Paulo, SP, Brazil; b Dermatology Discipline, Department of Internal Medicine, Irmandade da Santa Casa de Misericórdia de São Paulo, São Paulo, SP, Brazil

Dear Editor,

Lentigo maligno (LM) is a slow-growing, *in-situ* radial growth melanoma in which the neoplastic cells are confined to the epidermis and adnexal epithelium without surpassing the basal layer of these structures.^1^ LM represents 79% to 83% of cases of melanoma *in situ*, mainly affecting areas of intense sun damage, preferably the head and neck, with a peak incidence between 65 and 80 years of age and may become invasive in 5% to 15% of cases.^2^, ^3^

The histopathological analysis is the reference method for the diagnostic confirmation of LM, with the presence of a high number of atypical melanocytes in the basal layer of the epidermis and peri-adnexal extension associated with solar elastosis and epidermal thinning, due to the intense solar damage of the areas where these lesions originate.^3^, ^4^

One of the difficulties in the treatment of LM is to define the true clinical and histopathological lesion extension, as they present extended areas, that is, they can extend beyond the clinically visible margin, determining sometimes equivocal and/or unaesthetic margins in the surgical approach. Moreover, the recurrence rate described in the literature, according to the method used for lesion excision and the time of post-treatment follow-up, can reach 50%.^2^, ^4^, ^5^

Imiquimod (IQ) is an imidazoquinoline that acts on atypical melanocytes present in the epidermis, inducing an immune response through cytokine secretion and cell response against these tumor cells, which has been studied as a less invasive therapeutic option in the treatment of LM.^6^, ^7^

The topical use of Imiquimod 5%, applied six times a week for ten to 12 weeks, with a time of drug action of eight hours at the lesion site, results in clinical resolution in 78% of cases and histopathological resolution in 76%, with an average recurrence rate of 24.5%.^3^, ^6^, ^7^, ^8^

Aiming to increase the diagnostic accuracy and therapeutic control of LM, especially after less invasive alternative therapies, the use of laser reflectance confocal microscopy (RCM) is a non-invasive exam that allows the *in vivo* observation of skin structures and cells, with a similar resolution to that of histology.^3^, ^9^

RCM is reported to present an 85% sensitivity and 76% specificity for the diagnosis of LM.^4^ The increased number of atypical melanocytes at the dermal-epidermal junction, the presence of pagetoid cells and/or atypical cells in the epidermis, perifollicular dendritic cells, and nucleated cells in the dermal papilla are microscopic diagnostic criteria of ML on RCM.^3^, ^10^

These data stimulated the development of a descriptive observational study aiming at diagnosing, verifying the therapeutic result, and the early detection of possible recurrence through RCM of cases of LM, treated with topical Imiquimod 5% from March 2019 to July 2020.

Patients with a diagnosis of lentigo maligno confirmed by histopathological examination underwent 12 weeks of treatment with Imiquimod 5% cream with daily application, 6 times a week, with a time of drug action of eight hours.

Clinical and dermoscopic images of the lesions were obtained using an iPhone 10 coupled to Dermlite®; and RCM images were obtained with the aid of Vivascope 1500®, before, during, and 60 days after the end of the therapy. Cross-sectional 5 × 5 mm images of the stratum corneum, stratum granulosum, stratum spinosum, and superficial dermis were analyzed by two dermatologists with experience in performing this technique. Additionally, the RCM will be repeated every three months in the first year of follow-up for early diagnosis of possible recurrence.

Six patients with an anatomopathological diagnosis of lentigo maligno were selected for the study. The patients were phototypes II to III according to the Fitzpatrick classification, with a history of previous intense sun exposure and a mean age of 75 years. The regions affected by the lesions were the face in three cases (50%), the back in one case (16.7%), and the scalp in two cases (33.3%).

Dermatological examination showed irregular brownish lesions in photo exposed areas with intense sun damage, and dermoscopy showed an atypical pseudo network (Fig. 1). RCM showed the presence of atypical pagetoid cells in the epidermis and atypical perifollicular dendritic cells, with a loss of the epidermal structure (Fig. 2).

**Figure 1:**
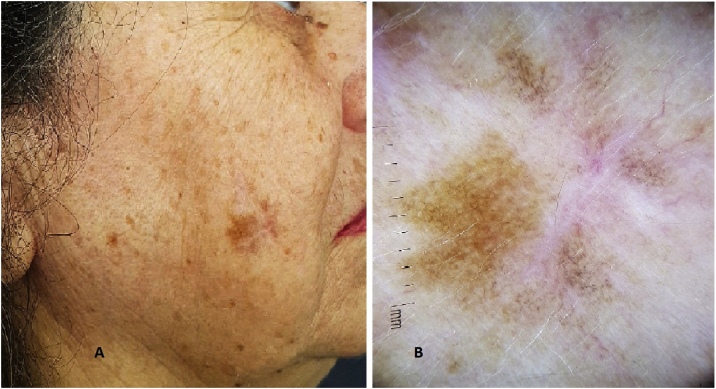
(A), Image of an asymmetric, brownish pigmented lesion on the face of a patient with sun photodamage. In the center of the lesion, a linear scar from a previous biopsy can be observed. (B), Dermoscopy of the lesion showing the presence of atypical pseudonetwork and areas of “peppering”.

**Figure 2:**
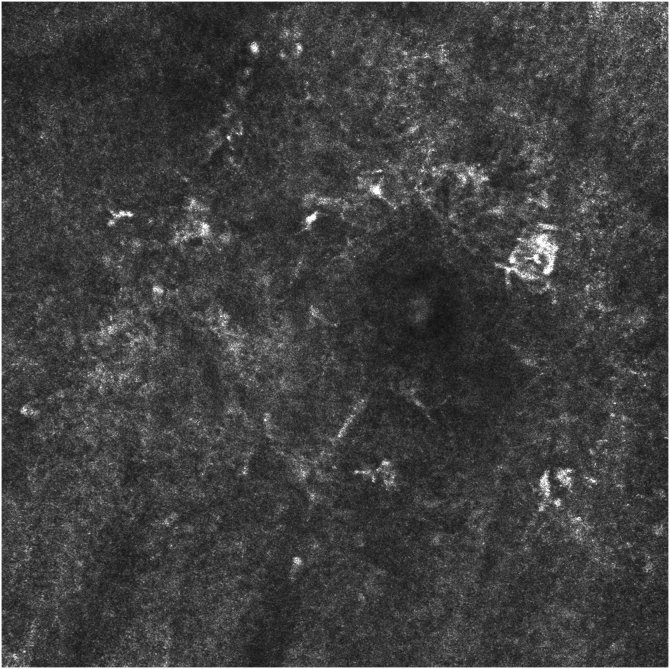
RCM image showing the presence of atypical pagetoid cells in the epidermis and atypical perifollicular dendritic cells, with loss of the epidermal structure.

Of the six selected patients, five completed 12 weeks of treatment with IQ 5%, and one patient was lost to follow-up at eight weeks of treatment. After 60 days of the end of treatment with IQ 5%, four patients underwent RCM, and one patient is scheduled to undergo post-treatment RCM. At the post-treatment dermatological examination, evidence of clinical and dermoscopic healing of the lesions was observed (Fig. 3), whereas RCM examination showed the absence of pagetoid and/or atypical cells in the epidermis, absence of perifollicular dendritic cells and a preserved epidermis (Fig. 4).

**Figure 3:**
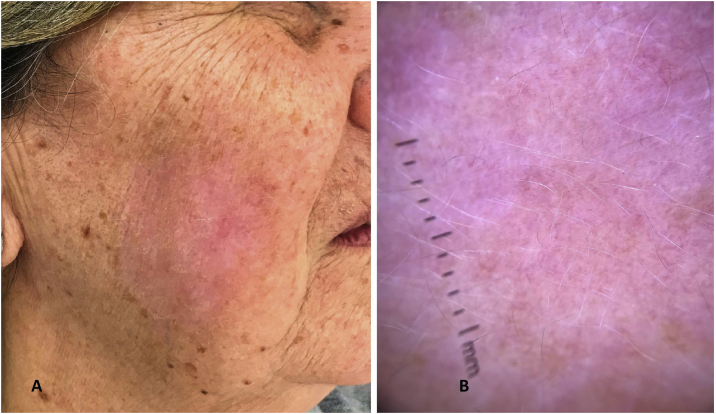
(A), Image of the clinical resolution of LM on the face after 12 weeks of treatment with topical IQ 5%. (B), Dermoscopy of the treated region showing a whitish pink background with a post-inflammatory characteristic.

**Figure 4:**
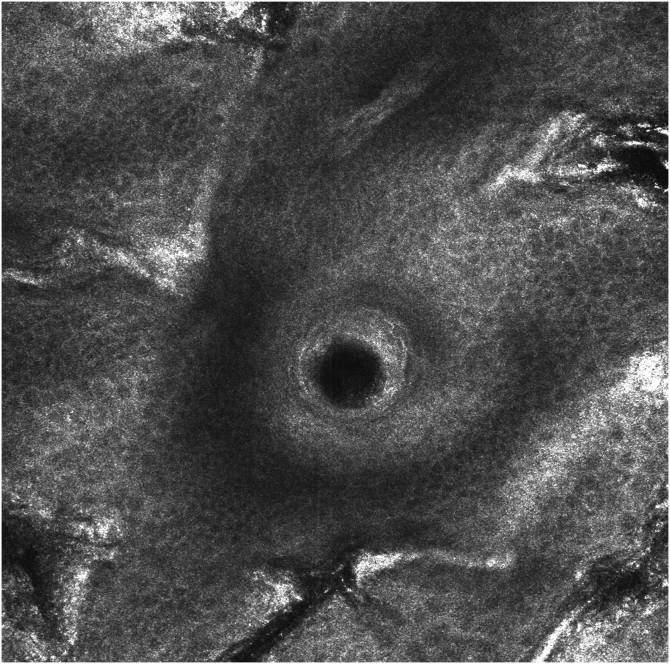
RCM image, 60 days after treatment with topical IQ 5% on the face, showing absence of atypical pagetoid cells in the epidermis and perifollicular dendritic cells, with preserved epidermal structure.

One of the patients, before participating in the study, had undergone three sessions of cryotherapy for the treatment of LM on the scalp, with clinical and dermoscopic resolution of the lesion. Four months after the last cryotherapy session, there was a recurrence of the lesion, diagnosed through RCM, with the appearance of atypical perifollicular dendritic cells in clinically and dermoscopically healed areas. The patient was then submitted to treatment with Imiquimod for 12 weeks, and the post-treatment RCM was performed, showing resolution of the condition.

The present study demonstrated that RCM allowed the early identification of recurrence of LM clinically and dermoscopically healed.

The present study is a work in progress, with preliminary data suggesting the use of RCM as a tool in the therapeutic control and follow-up of lentigo maligno treated with Imiquimod. LM affects mainly the elderly, presenting as extensive lesions of difficult clinical delimitation in areas such as the face and neck, which can result in difficulties regarding the surgical approach, with aesthetic damage to the patient. As it is an *in situ* lesion, it allows the use of less invasive treatments, and RCM has shown to be an extremely useful and practical tool in the therapeutic control and early diagnosis of possible recurrence. Better conclusions will be attained with longer follow-up periods and the inclusion of more cases. This prior communication is also an invitation to other authors to make similar observations.

## Financial support

None declared.

## Authors’ contributions

Priscila Ishioka: Approval of the final version of the manuscript; design and planning of the study; drafting and editing of the manuscript; collection, analysis, and interpretation of data; effective participation in research orientation; intellectual participation in the propaedeutic and/or therapeutic conduct of the studied cases; critical review of the literature; critical review of the manuscript.

Lilian Lemos Costa: Approval of the final version of the manuscript; drafting and editing of the manuscript; design and planning of the study; collection, analysis, and interpretation of data; intellectual participation in the propaedeutic and/or therapeutic conduct of the studied cases; critical review of the literature; critical review of the manuscript.

Marcus Maia: Approval of the final version of the manuscript; design and planning of the study; drafting and editing of the manuscript; collection, analysis, and interpretation of data; effective participation in research orientation; intellectual participation in the propaedeutic and/or therapeutic conduct of the studied cases; critical review of the literature; critical review of the manuscript.

## Conflicts of interest

None declared.
